# Supplementary material for: Most Earth-surface calcites precipitate out of isotopic equilibrium
Source: Nat Commun. 2019 Jan 25;10:429. doi: 10.1038/s41467-019-08336-5 (PMC6347637; doi:10.1038/s41467-019-08336-5)
Supplement: Supplementary file 1 — Supplementary Information [file 41467_2019_8336_MOESM1_ESM.pdf]

Supplementary Information for
"Most Earth-surface Calcites Precipitate Out of Isotopic Equilibrium"

(Daëron et al., 2019)

Date	*T* (°C)	pH	$HCO3^-$ (mg L^{-1})	Ca^{2+} (mg L^{-1})	Mg^{2+} (mg L^{-1})	Sr^{2+} (µg L^{-1})	Ba^{2+} (µg L^{-1})	U (µg L^{-1})	Mg/Ca (mol mol^{-1})	Sr/Ca (mmol mol^{-1})	Ba/Ca (mmol mol^{-1})	U/Ca (mmol mol^{-1})
22/05/2009	8.0	8.3	151.3	29.5	20.9	53	25	5.2	1.168	0.822	0.247	0.030
17/06/2009	7.3	8.4	157.4	30.2	21.0	55	28	5.1	1.146	0.833	0.271	0.028
28/07/2009	8.1	8.0	153.7	30.1	21.0	68	29	8.0	1.150	1.033	0.281	0.045
28/08/2009	8.0	8.4	154.4	29.7	20.7	65	28	7.9	1.149	1.001	0.275	0.045
02/10/2009	8.1	8.3	152.5	29.8	19.6	53	23	7.5	1.084	0.814	0.225	0.042
14/11/2009	7.9	8.2	143.4	28.0	17.5	54	23	7.9	1.030	0.882	0.240	0.048
14/12/2009	8.0	8.3	151.3	29.0	20.4	54	23	7.7	1.160	0.852	0.231	0.045
24/02/2010	8.0	8.2	164.7	29.9	21.8	57	24	6.4	1.202	0.872	0.234	0.036
27/03/2010	8.0	8.1	152.5	29.4	21.2	53	22	5.6	1.189	0.825	0.218	0.032
19/04/2010	7.9	8.4	154.4	28.5	21.0	53	23	5.5	1.215	0.851	0.236	0.032
31/05/2010	7.8	8.3	158.6	28.6	20.5	56	26	7.2	1.182	0.896	0.265	0.042
29/06/2010	7.8	8.3	149.5	30.0	20.8	58	25	6.9	1.143	0.884	0.243	0.039
05/08/2010	7.9	8.3	148.3	28.6	21.0	58	27	7.2	1.211	0.928	0.276	0.042
01/09/2010	7.9	8.3	148.3	28.9	20.8	56	24	7.0	1.187	0.886	0.242	0.041
30/09/2010	7.9	8.3	143.4	29.0	20.9	53	23	6.6	1.188	0.836	0.231	0.038
29/10/2010	7.9	8.2	146.4	27.1	20.8	58	24	7.1	1.265	0.979	0.258	0.044
25/11/2010	7.9	8.1	156.8	29.9	20.3	56	23	7.2	1.119	0.857	0.225	0.041
29/12/2010	7.9	8.1	149.5	29.3	21.0	56	22	7.1	1.182	0.874	0.219	0.041
03/02/2011	7.5	8.2	147.6	28.4	20.7	56	24	6.9	1.202	0.902	0.247	0.041
10/03/2011	8.0	8.2	154.4	29.4	19.8	55	22	6.6	1.110	0.856	0.218	0.038
05/04/2011	7.8	8.2	152.5	28.1	21.1	54	21	6.5	1.238	0.879	0.218	0.039
11/05/2011	7.7	8.2	148.3	28.5	21.4	56	22	6.5	1.238	0.899	0.225	0.038
24/06/2011	7.6	8.2	158.6	28.8	21.7	55	23	6.4	1.242	0.874	0.233	0.037
11/07/2011	7.8	8.1	148.9	29.1	21.4	55	23	6.3	1.212	0.865	0.231	0.036
09/08/2011	7.4	8.1	146.4	27.8	20.4	57	23	6.4	1.210	0.938	0.241	0.039
06/10/2011	7.9	8.2	151.3	29.7	21.8	56	22	6.3	1.210	0.862	0.216	0.036
07/11/2011	8.4	8.1	144.0	28.6	20.7	55	22	6.1	1.193	0.880	0.225	0.036
23/03/2012	8.1	7.9	151.3	32.0	20.7	52	21	5.7	1.067	0.743	0.192	0.030
Mean	7.9	8.2	151.4	29.1	20.7	56.0	23.8	6.7	1.175	0.879	0.238	0.039
2SD	0.4	0.2	10.0	1.8	1.6	6.9	4.2	1.6	0.116	0.116	0.042	0.010
1997–2006 observations (n=10, Piccini et al. *[31]):*												
Mean		8.2	154.0	30.3	20.6				1.121			
2SD		0.2	10.0	2.0	1.8				0.123			

Supplementary Table 1: Laghetto Basso pool water chemistry between 25/05/2009 and 23/03/2012. Along with the data previously published by *Piccini et al.* [31] (under the site name "Galleria Bassa"), the period of monitoring spans almost 15 years.

		Water		Calcite			
**Sample**	**N**	**T** (°C ± 1SE)	$\delta^{18}O_{VSMOW}$ (‰ ± 1SE)	$\delta^{13}C_{VPDB}$ (‰ ± 1SE)	$\delta^{18}O_{VPDB}$ (‰ ± 1SE)	Δ_{47} (‰ ± 1SE)	$^{18}\alpha_{cc/w}$
Adamussium colbecki	12	-1.8 ± 0.3	−0.5 ± 0.09	2.00 ± 0.04	4.45 ± 0.03	0.7715 ± 0.0067	1.0360
Neopycnodonte cochlear	20	13.5 ± 0.1	0.7 ± 0.2	0.83 ± 0.03	2.45 ± 0.02	0.7192 ± 0.0055	1.0327
Saccostrea cucullata	17	26.8 ± 0.9	1.7 ± 0.5	1.11 ± 0.03	−0.85 ± 0.03	0.6762 ± 0.0054	1.0283

Supplementary Table 2: Isotopic composition and water-calcite oxygen-18 fractionation factors for the bivalve samples.
